# Assessing the longitudinal association between the GGT/HDL-C ratio and NAFLD: a cohort study in a non-obese Chinese population

**DOI:** 10.1186/s12876-022-02598-y

**Published:** 2022-12-05

**Authors:** Qiyang Xie, Song Lu, Maobin Kuang, Shiming He, Changhui Yu, Chong Hu, Yang Zou

**Affiliations:** 1 Medical College of Nanchang University, Nanchang, 330006 China; 2 Jiangxi Cardiovascular Research Institute, Jiangxi Provincial People’s Hospital, The First Affiliated Hospital of Nanchang Medical College, Nanchang, 330006 China; 3 Gastroenterology Department, Jiangxi Provincial People’s Hospital, The First Affiliated Hospital of Nanchang Medical College, Nanchang, 330006 China

**Keywords:** Nonlinearity, Risk threshold, GGT/HDL-C ratio, Non-obese, Saturation threshold

## Abstract

**Background:**

A cross-sectional association between the combination indicator of high-density lipoprotein cholesterol (HDL-C) and gamma-glutamyl transferase (GGT) and fatty liver has been described in several recent studies, and this study aims to further evaluate the longitudinal relationship between the ratio of GGT to HDL-C (GGT/HDL-C ratio) and nonalcoholic fatty liver disease (NAFLD).

**Methods:**

This cohort study included 12,126 individuals without NAFLD at baseline, followed prospectively for 5 years, and the endpoint of interest was new-onset NAFLD. The relationship of the GGT/HDL-C ratio with new-onset NAFLD and the shape of the association was assessed by Cox regression models and restricted cubic spline (RCS) regression, respectively. Time-dependent receiver operator characteristics (ROC) curves were constructed to evaluate the predictive value of GGT, HDL-C, GGT/HDL-C ratio and BMI for the occurrence of NAFLD at different time points in the future.

**Results:**

The prevalence of NAFLD was 72.46/1000 person-years during the 5-year follow-up period. Results of multivariate Cox regression analysis showed a positive association of the GGT/HDL-C ratio with new-onset NAFLD after adequate adjustment of the related confounding factors, and the degree of correlation was slightly higher than that of GGT, and further subgroup analysis found that this association was more significant in the population with elevated systolic blood pressure (SBP). In addition, we also found a nonlinear relationship of the GGT/HDL-C ratio with the risk of new-onset NAFLD using the RCS regression, where the saturation threshold was about 31.79 U/mmol. Time-dependent ROC analysis results showed that the GGT/HDL-C ratio was increasingly valuable in predicting NAFLD over time, and was better than HDL-C in predicting NAFLD in the early stage (1–3 years), but was not superior to BMI and GGT.

**Conclusions:**

In this large longitudinal cohort study based on a Chinese population, our results supported that the GGT/HDL-C ratio was positively and nonlinearly associated with the risk of new-onset NAFLD in a non-obese population. In the assessment of future NAFLD risk, the GGT/HDL-C ratio was slightly better than GGT alone; However, the GGT/HDL-C ratio did not appear to have a significant advantage over GGT and BMI alone in predicting NAFLD.

**Supplementary Information:**

The online version contains supplementary material available at 10.1186/s12876-022-02598-y.

## Background

NAFLD is a group of progressive diseases including steatosis with or without mild inflammation, nonalcoholic steatohepatitis and cirrhosis characterized by accelerated progression of necroinflammation and fibrosis [1, 2]. NAFLD not only causes serious harm to the liver, but also adversely affects other organ systems in the human body [3], such as the increased risk of hepatocellular carcinoma, type 2 diabetes, sleep apnea, cardiovascular-related diseases, and chronic kidney disease [4, 5]. In addition, it’s noteworthy that with the changes in lifestyle and diet, the prevalence of NAFLD has increased rapidly; recent epidemiological analyses have shown that the prevalence of NAFLD is currently about 25% worldwide [1] and is an absolute risk factor for end-stage liver disease and the most common indication for liver transplantation [6, 7].

Obesity has been considered one of the most important risk factors for the increased prevalence and progression of NAFLD [8–10]. However, with the deepening of research in recent years, more and more researchers have found that non-obese people are also susceptible to NAFLD. Several recent epidemiological surveys showed that the global prevalence of non-obese NAFLD is currently between 14.5 and 15.75%, and it is more common in Asian populations, showing an overall upward trend [4, 11–13]. Furthermore, compared with obese NAFLD patients, some researchers believe that non-obese NAFLD patients usually have a worse prognosis and develop severe liver disease more quickly [4, 14]. Therefore, we believed that individuals with non-obese NAFLD need more attention and that special analysis and attention should be paid to important risk factors in this specific population.

GGT, a liver enzyme, is widely used as an indicator of liver function [15, 16] and is an important biomarker for identifying NAFLD [17]. HDL-C, known as the "good cholesterol", can play the role of antioxidant, anti-inflammatory, anti-thrombotic, promoting fibrinolysis, and improving endothelial function [18, 19], and is usually measured at lower concentrations in NAFLD patients [20–22]. In several recent cross-sectional studies specifically combining GGT with HDL-C [22, 23], researchers found that the GGT/HDL-C ratio was closely related to fatty liver (both metabolic and nonalcoholic) and significantly improved the ability of a single indicator to identify fatty liver; these findings suggested that the GGT/HDL-C ratio may be a useful marker for predicting NAFLD. However, it is unclear whether the correlation between GGT/HDL-C ratio and NAFLD will change over time, whether a similar association exists in non-obese people, and whether there is a nonlinear association between the two. To clarify the answers to these questions, the current study based on a large longitudinal cohort in China aimed to further explore the relationship between NAFLD and the GGT/HDL-C ratio in non-obese individuals.

## Methods

### Research population

This study is a secondary analysis of a recent cross-sectional and longitudinal study conducted by Wenzhou People’s Hospital and the use of data from the study has been authorized by the original data author, Professor Zheng, and his team. Detailed research design, organization and implementation have been reported elsewhere [24]. Briefly, in the initial study, Zheng et al. conducted a cross-sectional study and a longitudinal study at the Medical examination Center of Wenzhou people’s Hospital from January 2010 to December 2014; the cross-sectional study included 183,903 non-obese individuals who underwent health screening, and the longitudinal study included 16,173 individuals with 5-year follow-up data (Additional files 1 and 2). In the current study, we analyzed mainly based on the longitudinal cohort in this data set, excluding people with excessive drinking habits, diagnosed with liver disease, on medication at baseline, baseline low-density lipoprotein cholesterol (LDL-C) > 3.12 mmol/L, missing follow-up data, baseline body mass index (BMI) ≥ 25 kg/m^2^, and missing baseline data for GGT and HDL-C based on the new study hypothesis, and we finally included 12,126 participants for study analysis. In addition, we also validated the ROC results of previous studies based on cross-sectional data of this dataset.

### Ethical approval and consent to participate

In the original study, the investigators obtained verbal informed consent from each subject and Wenzhou People’s Hospital ethics committee approved the previous research protocol. The current research didn’t require another application for informed consent and ethical approval because it was a secondary analysis of the previous research and the identifying information of the subjects had been removed from the study dataset. The entire process of this research followed the Declaration of Helsinki.

### Data acquisition

Baseline characteristics of subjects were recorded by trained medical staff at the physical examination center. Blood pressure was measured by a noninvasive sphygmomanometer (OMRON, Japan) in a quiet environment; height and weight were measured with the subject’s shoes and coat removed, and BMI was calculated for each subject.

Venous blood samples were drawn from the anterior elbow vein under fasting, and parameters such as creatinine (CR), aspartate aminotransferase (AST), fasting plasma glucose (FPG), blood urea nitrogen (BUN), total protein, uric acid (UA), albumin, globulin, total cholesterol (TC), alkaline phosphatase (ALP), total bilirubin, triglyceride (TG), direct bilirubin, HDL-C, alanine aminotransferase (ALT), LDL-C and GGT were measured by an automated analyzer (Abbott AxSYM) using standardized methods in laboratories certified by international organizations.

### Evaluation of IR (insulin resistance)

In the current study, we calculated the metabolic score for the IR index (MetS-IR) as an alternative to IR. MetS-IR was calculated as (ln ((2 × FPG) + TG) × BMI)/(ln (HDL-C)) [25].

### Follow-up and diagnosis of NAFLD

After the baseline assessment visit, subjects were assessed annually by abdominal ultrasonography and prospectively followed for 5 years, with the primary endpoint being the diagnosis of new-onset NAFLD. The diagnosis of NAFLD referred to the criteria recommended by the Chinese Society of Liver Diseases [26], which mainly include the following five items: (1) poor or incomplete visualization of the right hepatic lobe and diaphragmatic capsule; (2) decreased hepatic blood flow signal; (3) mild to moderate hepatomegaly with rounded edges; (4) the intrahepatic cavity structure is unclear; (5) the near-field echo of the liver area is diffusely enhanced, and the far-field echo is gradually weakened.

### Statistical analysis

Baseline characteristics description: The GGT/HDL-C ratio was divided into five equal parts by the quantile function to describe the baseline characteristics of the subjects and the chi-square test or one-way ANOVA or Kruskal–Wallis H test was selected to compare the differences between groups according to the type and distribution pattern of the variables.

Assumptions of the Cox regression model: Before establishing the Cox regression model, we first assessed whether the assumption of log-linearity was satisfied between the independent variables and covariates and whether the assumption of proportional risk was satisfied between the independent variables and the dependent variable. (1) We constructed Kaplan–Meier curves for the incidence of NAFLD corresponding to quintile groupings of GGT/HDL-C ratio, and judged whether the assumption of equal proportional risk was violated by assessing whether there was a crossover of the curves (Additional file 3: Figure S1); (2) through multiple linear regression we examined the linear relationships between the covariates and calculated the variance inflation factors for each covariate (Additional file 4: Table S1).

Correlation analysis: according to the recommendations of the STROBE statement, we constructed 5 Cox regression models for assessing the longitudinal relationship of NAFLD with the GGT/HDL-C ratio and recorded the corresponding Hazard ratio (HR) and 95% confidence interval (CI) [27]. Among the five models, the crude model was a simple model without adjustment for variables, and model I was initially adjusted for demographic characteristics (including age, height, BMI and sex); model II further considered the effect of liver and kidney function on NAFLD (ALP, AST, ALT, and Cr) based on the model I; model III was further adjusted for factors related to blood glucose, blood lipids, and blood pressure; model IV additionally adjusted ALB, GLB, UA and BUN on the basis of Model III. In these five models, we also examined the trend between the median of each GGT/HDL-C ratio quintile and the NAFLD risk to verify the stability of the direction of the association. In order to further evaluate the ability of GGT/HDL-C ratio and its components (GGT, HDL-C) and BMI to assess and predict the risk of future NAFLD, we used standardized HR to show the correlation of baseline GGT, HDL-C, GGT/HDL-C ratio and BMI with future NAFLD based on the final model (model IV). Furthermore, we also used R-packet timeROC to construct ROC curves at 5 follow-up time points; then to calculate the area under the ROC curves (AUCs) for each parameter from year 1 to year 5 and record the corresponding sensitivity/specificity to judge the predictive value of GGT/HDL-C ratio, GGT, HDL-C and BMI to the future NAFLD. On the other hand, we also performed ROC analysis in this study using the cross-sectional study data of the dataset, aiming to verify whether the diagnostic value of GGT/HDL-C ratio for NAFLD in the population is stable. In addition, we performed a mediation analysis to determine whether the effect of the GGT/HDL-C ratio on NAFLD was mediated by MetS-IR, and quantified the magnitude of the mediation effect by calculating the ratio of indirect effects to total effects to obtain the percentage of mediation.

Subgroup analyses: We performed several subgroup analyses to evaluate the longitudinal association between the GGT/HDL-C ratio and NAFLD in populations of different ages, sex, blood pressure, and glycemic metabolic status.

Nonlinear analysis: we also used the RCS nested in the Cox regression Model IV with 4-knot for fitting the shape of the dose–response relationship of the GGT/HDL-C ratio with NAFLD risk and estimated the corresponding saturation thresholds.

All data in this study were analyzed using R statistical software (V3.4.3) and Empower (R, V2.0). All *P* values were two-sided, and *P* < 0.05 was considered statistically significant.

## Results

### Baseline characteristics

This study included 12,126 non-obese subjects (mean age 43.3 years, 5485 women and 6524 men) who met the inclusion criteria, with a prevalence of NAFLD of 72.46/1000 person-years during the 5-year follow-up period. Table 1 shows the basic characteristics of the subject population according to GGT/HDL-C ratio quintiles, and we found that the vast majority of clinical baseline characteristics increased with the increasing GGT/HDL-C ratio quintiles, while several of the lipid-related indicators (TC, TG, HDL-C) showed opposite trends.

**Table 1:** Baseline characteristics of five groups

	GGT/HDL-C ratio quintile					*P*-value
	Q1 (1.40–6.92)	Q2 (6.93–8.75)	Q3 (8.75–11.16)	Q4 (11.16–16.15)	Q5 (≥ 16.16)	
No. of participants	2425	2424	2426	2425	2426	
Sex						**<** 0.001
Women	1187 (48.95%)	1144 (47.19%)	1101 (45.38%)	1055 (43.51%)	995 (41.01%)	
Men	1238 (51.05%)	1280 (52.81%)	1325 (54.62%)	1370 (56.49%)	1431 (58.99%)	
Age, years	40.00 (32.00–51.00)	40.00 (31.00–51.00)	40.00 (31.00–51.00)	41.00 (32.00–53.00)	41.00 (32.00–53.00)	0.015
Height, m	1.63 ± 0.07	1.65 ± 0.08	1.66 ± 0.08	168 ± 0.08	168 ± 0.07	**<** 0.001
Weight, kg	56.50 ± 7.74	58.28 ± 8.28	59.73 ± 8.47	61.56 ± 8.55	63.49 ± 7.83	**<** 0.001
BMI, kg/m^2^	21.11 (19.78–22.49)	21.40 (19.79–22.90)	21.59 (20.00–23.14)	22.10 (20.47–23.49)	22.66 (21.19–23.84)	**<** 0.001
ALP, g/L	64.81 ± 18.18	68.74 ± 19.95	71.94 ± 20.36	74.55 ± 21.44	81.49 ± 29.25	**<** 0.001
GGT, U/L	14.00 (12.00–16.00)	18.00 (16.00–21.00)	22.00 (19.00–25.00)	28.00 (24.00–34.00)	51.00 (39.00–74.00)	**<** 0.001
ALT, U/L	13.00 (10.00–16.00)	14.00 (11.00–19.00)	16.00 (12.00–21.00)	18.00 (14.00–25.00)	24.00 (18.00–35.00)	**<** 0.001
AST, U/L	19.00 (17.00–22.00)	20.00 (17.00–23.00)	21.00 (18.00–24.00)	22.00 (19.00–26.00)	25.00 (22.00–31.00)	**<** 0.001
TP, g/L	73.33 ± 4.27	73.84 ± 4.14	74.04 ± 4.08	73.97 ± 4.38	74.08 ± 4.36	**<** 0.001
ALB, g/L	44.23 ± 2.76	44.50 ± 2.68	44.73 ± 2.66	44.65 ± 2.86	44.582 ± .87	**<** 0.001
GLB, g/L	29.10 ± 3.88	29.34 ± 3.80	29.323 ± .77	29.32 ± 4.19	29.50 ± 4.21	0.025
TB, μmol/L	10.60 (8.30–13.60)	11.10 (8.70–14.10)	11.50 (9.00–14.70)	11.70 (9.20–14.90)	11.80 (9.40–15.20)	**<** 0.001
DBIL, μmol/L	1.90 (1.30–2.50)	1.90 (1.40–2.60)	2.00 (1.50–2.70)	2.00 (1.50–2.70)	2.00 (1.40–2.80)	**<** 0.001
BUN, mmol/L	4.30 (3.50–5.20)	4.30 (3.58–5.22)	4.40 (3.68–5.37)	4.45 (3.70–5.30)	4.50 (3.80–5.40)	**<** 0.001
CR, mmol/L	73.00 (65.00–86.00)	77.00 (67.00–91.00)	83.00 (70.00–95.00)	86.00 (74.00–96.00)	88.00 (77.00–98.00)	**<** 0.001
UA, μmol/L	240.00 (195.00–304.00)	264.00 (210.00–329.00)	288.00 (231.00–346.00)	311.00 (250.20–364.00)	338.00 (280.00–393.00)	**<** 0.001
FPG, mmol/L	5.10 ± 0.66	5.11 ± 0.60	5.15 ± 0.71	5.27 ± 0.95	5.431 ± .10	**<** 0.001
TC, mmol/L	4.87 ± 0.63	4.66 ± 0.65	4.52 ± 0.70	4.45 ± 0.75	4.55 ± 0.88	< 0.001
TG, mmol/L	0.98 (0.77–1.29)	1.05 (0.80–1.38)	1.11 (0.82–1.51)	1.24 (0.91–1.72)	1.52 (1.08–2.27)	< 0.001
HDL-C, mmol/L	1.52 (1.30–1.76)	1.46 (1.24–1.71)	1.40 (1.18–1.66)	1.35 (1.13–1.61)	1.32 (1.10–1.58)	< 0.001
LDL-C, mmol/L	2.51 (2.23–2.80)	2.35 (2.02–2.65)	2.23 (1.92–2.58)	2.18 (1.82–2.54)	2.16 (1.76–2.53)	< 0.001
SBP, mmHg	118.411 ± 6.63	119.57 ± 16.19	121.82 ± 16.45	124.12 ± 16.80	128.11 ± 16.84	< 0.001
DBP, mmHg	71.11 ± 9.83	72.22 ± 9.85	73.65 ± 10.16	74.63 ± 10.18	77.43 ± 10.78	< 0.001

Values are n (%) or mean (standard deviation) or median (interquartile range)

*BMI* body mass index, *BUN* blood urea nitrogen, *Cr* creatinine, *UA* uric acid, *FPG* fasting plasma glucose, *TC* total cholesterol, *TG* triglyceride, *HDL-C* high-density lipoprotein cholesterol, *LDL-C* low-density lipoprotein cholesterol, *ALP* Alkaline phosphatase, *GGT* gamma-glutamyl transferase, *ALT* alanine aminotransferase, *AST* aspartate aminotransferase, *TP* Total Protein, *ALB* albumin, *GLB* globulin, *TB* Total bilirubin, *DBIL* Direct bilirubin, *DBP* diastolic blood pressure, *SBP* systolic blood pressure

### Correlation of the GGT/HDL-C ratio with NAFLD

Before performing multivariate Cox regression analysis, the data analyzed in the current research conformed to the assumption of equal proportional hazards and log-linearity. We constructed 5 Cox regression models according to the STROBE statement. In the crude model, the GGT/HDL-C ratio was positively correlated with NAFLD risk (HR: 1.11, 95% CI 1.10, 1.13), while in the adjusted model (models I–IV), the direction of this association was unchanged, and the degree of association was slightly weakened. Among them, the risk of new-onset NAFLD increased by 7% (HR: 1.07, 95% CI 1.04, 1.10) for each standard deviation increase in the GGT/HDL-C ratio in Model IV, and the HRs and 95% CIs for the lowest to highest quintiles of the GGT/HDL-C ratio were 1, 1.56 (1.28, 1.91), 2.20 (1.82, 2.67), 2.73 (2.26, 3.29), 3.38 (2.79, 4.08), respectively, showing a positive correlation trend (*P* for trend < 0.001) (Table 2). Based on model 4, we further evaluated the correlation between GGT, HDL-C and BMI and NAFLD (Table 3). After standardizing HR, we found that the standardized HR value of the GGT/HDL-C ratio was slightly higher than that of GGT and HDL-C, while the standardized HR value of BMI was higher than the GGT/HDL-C ratio in evaluating the risk of future NAFLD.

**Table 2:** Hazard ratios for NAFLD events by quintiles of GGT/HDL-C ratio

	HR (95% CI)						*P* for trend
	Multivariable analysis	Q1	Q2	Q3	Q4	Q5	
*GGT/HDL-C ratio (per SD)*							
Crude model	1.11 (1.10, 1.13)	Ref	1.68 (1.37, 2.05)	2.52 (2.09, 3.04)	3.52 (2.94, 4.21)	5.29 (4.45, 6.29)	< 0.001
Model I	1.09 (1.07, 1.10)	Ref	1.49 (1.22, 1.82)	2.11 (1.75, 2.55)	2.57 (2.14, 3.09)	3.45 (2.90, 4.12)	< 0.001
Model II	1.09 (1.06, 1.11)	Ref	1.47 (1.20, 1.79)	2.05 (1.70, 2.48)	2.51 (2.09, 3.01)	3.28 (2.74, 3.92)	< 0.001
Model III	1.08 (1.05, 1.10)	Ref	1.55 (1.27, 1.89)	2.18 (1.81, 2.64)	2.71 (2.25, 3.26)	3.33 (2.76, 4.01)	< 0.001
Model IV	1.07 (1.04, 1.10)	Ref	1.56 (1.28, 1.91)	2.20 (1.82, 2.67)	2.73 (2.26, 3.29)	3.38 (2.79, 4.08)	< 0.001

*NAFLD* nonalcoholic fatty liver disease, *CI* confidence interval, *HR* hazard ratios; other abbreviations as in Table 1; Crude model adjusted for none; Model I adjusted for sex, age, height and BMI; Model II adjusted for model 1 plus ALT, AST, ALP and Cr; Model III adjusted for model 2 plus FPG, TG, LDL, SBP and DBP; Model IV adjusted for model III plus ALB, GLB, UA and BUN

**Table 3:** Association of GGT, HDL-C, GGT/HDL-C ratio and BMI with NAFLD risk

	HR (95% CI)	*P*-value
HDL-C (per SD)	0.84 (0.79, 0.88)	< 0.0001
GGT (per SD)	1.06 (1.03, 1.10	< 0.0001
GGT/HDL-C ratio (per SD)	1.07 (1.04, 1.10	< 0.0001
BMI (per SD)	2.48 (2.32, 2.65)	< 0.0001

Adjusted for sex, age, height, BMI, ALT, AST, ALP, Cr, FPG, TG, LDL, SBP, DBP, ALB, GLB, UA and BUN

BMI itself is not adjusted in models with BMI as the independent variable

*CI* confidence interval, *HR* hazard ratios; other abbreviations as in Table 1

### Values of GGT, HDL-C, GGT/HDL-C ratio and BMI for diagnosis and prediction of NAFLD

Based on the cross-sectional data set of 183,903 non-obese individuals, we analyzed the diagnostic value of GGT, HDL-C, GGT/HDL-C ratio and BMI in NAFLD. The results showed that GGT/HDL-C ratio and BMI were comparable in identifying NAFLD (AUC: 0.8050 vs 0.8164), and both were better than GGT (AUC: 0.7780) and HDL-C (0.6986) alone (Additional file 4: Table S2).

Based on longitudinal data, we further calculated the AUC of GGT, HDL-C, GGT/HDL-C ratio and BMI at multiple time points by time-dependent ROC (Table 4). The results showed that the GGT/HDL-C ratio was increasingly valuable in predicting NAFLD over time, and was better than HDL-C in predicting NAFLD in the early stage (1–3 years), but was not superior to BMI and GGT.

**Table 4:** Best threshold and areas under the time-dependent receiver operating characteristic curves for BMI, WC and WHtR predicting future diabetes risk

	BMI		GGT		HDL-C		GGT/HDL-C ratio	
	AUC	Sensitivity (specificity)	AUC	Sensitivity (specificity)	AUC	Sensitivity (specificity)	AUC	Sensitivity (specificity)
1-Years	0.77	0.86 (0.59)	0.66	0.78 (0.50)	0.50	0.81 (0.24)	0.62	0.56 (0.62)
2-Years	0.76	0.80 (0.61)	0.67	0.73 (0.56)	0.54	0.77 (0.30)	0.63	0.64 (0.56)
3-Years	0.77	0.81 (0.61)	0.69	0.73 (0.59)	0.60	0.75 (0.43)	0.65	0.68 (0.57)
4-Years	0.78	0.81 (0.62)	0.69	0.71 (0.6)	0.70	0.68 (0.64)	0.65	0.72 (0.53)
5-Years	0.78	0.91 (0.53)	0.73	0.76 (0.66)	0.74	0.81 (0.65)	0.70	0.81 (0.52)

*AUC* area under the curve; other abbreviations as in Table 1

### Subgroup analysis

We also analyzed the NAFLD risk related to GGT/HDL-C ratio in different ages, sexes, and FPG, SBP, and DBP levels, and compared the difference between different stratifications to determine whether there was an interaction with GGT/HDL-C ratio. The results of the research showed that we only observed a significant interaction between the GGT/HDL-C ratio and SBP in the subgroup (*P*-interaction = 0.0361), where the NAFLD risk related to GGT/HDL-C ratio was significantly higher in those with SBP ≥ 140 mmHg (Table 5).

**Table 5:** Stratified associations between GGT/HDL-C ratio and NAFLD by age, sex, FPG, SBP and DBP

	No.of cases	HR (95% CI)	*P* for interaction
*Age (years)*			0.2383
≤ 30	349	1.12 (1.02, 1.22)	
> 30, ≤ 45	843	1.04 (0.99, 1.09)	
> 45, ≤ 60	601	1.09 (1.04, 1.15)	
≥ 60	354	1.11 (1.02, 1.20)	
*Sex*			0.5629
Men	1210	1.07 (1.04, 1.10)	
Women	937	1.05 (1.00, 1.11)	
*FPG, mmol/L*			0.6719
≥ 6.1	293	1.05 (0.96, 1.15)	
< 6.1	1854	1.07 (1.04, 1.10)	
*SBP, mmHg*			0.0361
< 140	1677	1.05 (1.02, 1.09)	
≥ 140	469	1.14 (1.07, 1.21)	
*DBP, mmHg*			0.4003
< 90	1819	1.09 (1.05, 1.13)	
≥ 90	327	1.06 (1.01, 1.12)	

*DBP* diastolic blood pressure, *SBP* systolic blood pressure, *FPG* fasting plasma glucose, *CI* confidence interval, *HR*: hazard ratios

### Nonlinear analysis

**Fig. 1:**
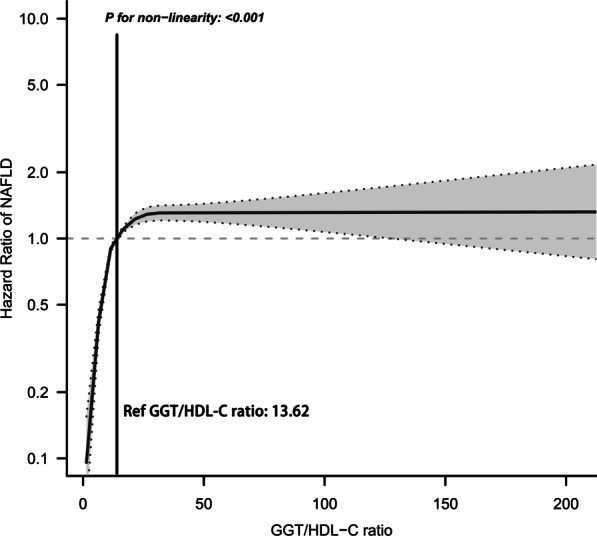
Hazard ratios (95% confidence intervals) for the nonlinear relationship between the GGT/HDL-C ratio and the risk of NAFLD. Adjusted for sex, age, ALP, GGT, ALT, AST, ALB, GLB, CR, UA, FPG, TC, TG, height, BMI, SBP, DBP, BUN and UA; NAFLD: nonalcoholic fatty liver disease. *GGT* gamma-glutamyl transferase, *HDL-C* high-density lipoprotein cholesterol

We fitted dose–response curves for the relationship of the GGT/HDL-C ratio with NAFLD risk by RCS. As shown in Fig. 1, the association of NAFLD and the GGT/HDL-C ratio was nonlinear and positive. Based on the dose–response curve, we found that the risk of NAFLD no longer increased when the GGT/HDL-C ratio was greater than 31.79 U/mmol.

### Mediation analysis

Table 6 shows the results of the mediation analysis of MetS-IR in the relationship between GGT/HDL-C ratio and NAFLD. In the non-obese population, we found that MetS-IR partially mediated the association between GGT/HDL-C ratio and NAFLD risk, with the intermediary accounting for 12.8%.

**Table 6:** Mediated analysis of GGT/HDL-C ratio and NAFLD by MetS-IR

Mediator	Total effect	Mediation effect	Direct effect	PM (%)	*P*-value of PM
MetS-IR	0.01346 (0.00956, 0.01778)	0.00172 (0.00105, 0.00248)	0.01174 (0.00790, 0.01593)	12.8	< 0.001

Adjusted for sex, age, height, BMI, ALT, AST, ALP, Cr, FPG, TG, LDL, SBP, DBP, ALB, GLB, UA and BUN

*MetS-IR* metabolic score for insulin resistance, *PM* propotion mediate

## Discussion

The main findings of this longitudinal cohort study based on the non-obese Chinese population were as follows: (1) GGT/HDL-C ratio was positively correlated with the NAFLD risk among Chinese non-obese people, and the association was stronger in those with elevated SBP. (2) GGT/HDL-C ratio had a nonlinear relationship with NAFLD, and there was a saturation effect. (3) GGT/HDL-C ratio and BMI were comparable in identifying NAFLD and were superior to GGT and HDL-C alone; however, GGT/HDL-C ratio did not appear to have a significant advantage in predicting future NAFLD.

With the change in diet and lifestyle in recent decades, the prevalence of non-obese NAFLD is increasing all over the world [11–13, 28], and research on non-obese NAFLD is also increasing [13, 29]. According to some previous research evidence, non-obese patients with NAFLD tended to exhibit worse metabolic status than obese patients with NAFLD, and they may develop more severe non-alcoholic steatohepatitis and advanced fibrosis [14, 30, 31]. Moreover, related studies have also shown that non-obese NAFLD had a higher risk of developing metabolic syndrome and diabetes [12, 32], and a higher risk of all-cause mortality [33]. These findings all suggested the specificity of non-obese NAFLD and that this population needs further attention.

GGT is the most commonly used clinical biochemical index to evaluate liver function, and in addition to identifying the risk of NAFLD prevalence [15–17], it is also closely associated with NAFLD-related complications [34]. HDL is a multifunctional structural protein, and HDL functionality represents several performance metrics of HDL, such as antioxidant, anti-inflammatory, and cholesterol efflux activities [35, 36]. Previous studies have shown that the emergence of dysfunctional HDL is usually associated with many acute infectious diseases and chronic aging-related diseases. HDL can be an appropriate biomarker for the diagnosis and progression of many diseases by monitoring the changes in its quantity and quality in terms of antioxidant and anti-inflammatory capacity [36]. It is worth noting that in several recent studies on the relationship between HDL function and NAFLD, researchers have shown that the imbalance of cholesterol efflux activity in HDL function may be the main cause of metabolic NAFLD [37, 38]. The quantity of HDL is expressed as serum HDL-C concentration, and low concentration of HDL-C is a common clinical lipid metabolism disorder [36, 39], which is closely related to the progressive course of NAFLD [20–22, 40]. In the current study, after dividing and combining these two indicators, we found that the ratio of GGT/HDL-C was positively related to NAFLD risk in the Chinese non-obese population, and the positive association between them existed stably after further variable adjustment, and the extent of the association changed only slightly. Studies on the association of NAFLD with the GGT/HDL-C ratio have also been described previously. As early as May 2020, Feng et al. first revealed a positive relationship of NAFLD with the GGT/HDL-C ratio through a cross-sectional study of 6,326 general population [22]; then in a recent study by Xing et al. of 1,434 inpatients with diabetes, they found that the GGT/HDL-C ratio was an independent risk factor for metabolic-associated fatty liver disease in people with diabetes with a BMI ≥ 23 kg/m^2^ [23]. Although both studies found a relationship of NAFLD with the GGT/HDL-C ratio, it is unclear whether the relationship of the GGT/HDL-C ratio with NAFLD changes over time and whether there is a nonlinear association between the two due to the cross-sectional design. Therefore, we conducted the current study, and our results further revealed the longitudinal correlation between GGT/HDL-C ratio and NAFLD. Additionally, we further evaluated baseline GGT/HDL-C ratio, GGT, HDL-C, and BMI for correlation with future NAFLD and used standardized HR values to present all results; the study found that GGT/HDL-C ratio may be slightly better than GGT, and BMI was better than GGT/HDL-C ratio in assessing future NAFLD risk.

The diagnostic value of GGT, HDL-C and GGT/HDL-C ratio for NAFLD was previously described by Feng et al. Through ROC analysis, they found that the diagnostic value of GGT combined with HDL-C for NAFLD was significantly higher than that of GGT and HDL-C alone [22]. In order to verify their findings, we have carried out the same analysis in the current cross-sectional data sets, and the results were consistent with the findings of Feng et al. We found that the ratio of GGT/HDL-C was similar to BMI but superior to GGT and HDL-C alone in the diagnosis of NAFLD. In addition, in the current study, we further carried out time-dependent ROC analysis based on longitudinal data. The results show that with the passage of time, although the ratio of GGT/HDL-C seemed to be increasingly valuable in predicting NAFLD and was better than HDL-C in the early stage, it was not superior to BMI and GGT. Taken together, the GGT/HDL-C ratio seemed to be more suitable for epidemiological screening of NAFLD than for prediction of NAFLD, and further studies are still needed to validate the findings based on longitudinal data.

In the current longitudinal cohort, we also examined whether there were differences in the NAFLD risk associated with the GGT/HDL-C ratio in those of different ages, sex, FPG, SBP, and DBP populations. The results showed that the NAFLD risk associated with the GGT/HDL-C ratio was only significant in those with high SBP, while no significant differences were observed in the stratification of DBP, ages, sex, and FPG. It is well known that SBP is an important risk factor for NAFLD, even when elevated within normal levels [41, 42]. We, therefore, suggested that the higher risk of NAFLD associated with the GGT/HDL-C ratio in those with high SBP may be related to the common pathophysiological mechanisms of hypertension and NAFLD. According to the literature, patients with high levels of SBP and those with NAFLD both were more likely to develop insulin resistance (IR), and vascular and adipose tissue inflammation [43].

Notably, this study also found a nonlinear association of the GGT/HDL-C ratio with NAFLD. Through the dose–response relationship curve, we estimated a saturation threshold of approximately 31.79 U/mmol; when the GGT/HDL-C ratio exceeded the saturation threshold, the risk of developing NAFLD was no longer increased. These new findings provided useful intervention thresholds for the prevention of NAFLD, and to my knowledge, this study is the first to reveal a nonlinear association between the GGT/HDL-C ratio and NAFLD.

The mechanism underlying the correlation between the GGT/HDL-C ratio and new-onset NAFLD risk is unclear, and may be partially explained by the following two reasons: (1) According to the "three strikes theory", the main pathological changes in the pathogenesis of NAFLD and its pathological progression include steatosis, lipotoxicity, and chronic inflammation [2]; and high levels of GGT are associated with both hepatic steatosis and chronic inflammation [34, 44]; Additionally, lipid disorders, high levels of GGT accompanying low levels of HDL-C, often lead to an increase in blood fatty acids, which are converted to excess TG when the blood fatty acids exceed the tolerance processing capacity of adipose tissues and various tissues, subsequently causing further organ damage, i.e., lipotoxicity [2]. (2) It is well known that IR is the main pathophysiological mechanism of NAFLD [45]. In order to explore the important role of IR in the relationship between GGT/HDL-C ratio and NAFLD, an mediation analysis was carried out in this study. The findings confirmed that MetS-IR played an intermediary role in the relationship between GGT/HDL-C ratio and NAFLD. These findings provided useful evidence for further revealing the relationship between GGT/HDL-C ratio and NAFLD.

The GGT/HDL-C ratio is an easily accessible clinical parameter, and the results of this study provided useful evidence for risk screening of NAFLD in non-obese individuals. On the basis of the current research results, we put forward several meaningful suggestions for a series of work that may be carried out in the future: (1) According to our results, we should pay more attention to the evaluation of GGT/HDL-C ratio of non-obese individuals, especially those with high SBP. (2) Non-obese people with a high GGT/HDL-C ratio found in clinical practice or routine physical examination should be offered appropriate interventions to prevent NAFLD, such as weight loss and improved dietary patterns [46].

### Limitations

Some limitations are worth noting: (1) the findings of this study can be applied to the non-obese population in China, but their applicability in other countries and ethnic populations is unclear; (2) the diagnosis of NAFLD in the current study was not performed by liver biopsy, which may increase the false-negative rate of new-onset NAFLD diagnosis, causing the incidence in the study to be lower than the true situation; (3) as with other observational studies, there were still unadjusted covariates that were not measurable or not found (Additional files 3, 4).

## Conclusion

In a word, in this longitudinal cohort study of non-obese people in China, we found that GGT/HDL-C ratio was positively correlated with new-onset NAFLD, and this independent risk association was further increased in people with high SBP. Additionally, in the assessment of future NAFLD risk, the GGT/HDL-C ratio was slightly better than GGT alone; However, the GGT/HDL-C ratio did not appear to have a significant advantage over GGT and BMI alone in predicting NAFLD.

## Supplementary Information

**Additional file 1.** Cross-sectional data sets for research.

**Additional file 2.** Longitudinal data set for research.

**Additional file 3.** Supplementary Figure.

**Additional file 4.** Supplementary Table.

## Data Availability

The dataset used in this study has been authorized by Professor Zheng and his team and has been uploaded to the system as a Additional files 1, 2, 3 and 4.

## Abbreviations

NAFLD: Nonalcoholic fatty liver disease
HDL-C: High-density lipoprotein cholesterol
GGT: Gamma-glutamyl transferase
RCS: Restricted cubic spline
HR: Hazard ratio
CI: Confidence interval
SBP: Systolic blood pressure
BMI: Body mass index
LDL-C: Low-density lipoprotein cholesterol
ALP: Alkaline phosphatase
ALT: Alanine aminotransferase
AST: Aspartate aminotransferase
BUN: Blood urea nitrogen
Cr: Creatinine
UA: Uric acid
FPG: Fasting plasma glucose
TC: Total cholesterol
TG: Triglyceride
IR: Insulin resistance
