# Supplementary material for: Assessing the longitudinal association between the GGT/HDL-C ratio and NAFLD: a cohort study in a non-obese Chinese population
Source: BMC Gastroenterol. 2022 Dec 5;22:500. doi: 10.1186/s12876-022-02598-y (PMC9724423; doi:10.1186/s12876-022-02598-y)
Supplement: Supplementary file 3 — Additional file 3. Supplementary Figure. [file 12876_2022_2598_MOESM3_ESM.pdf]

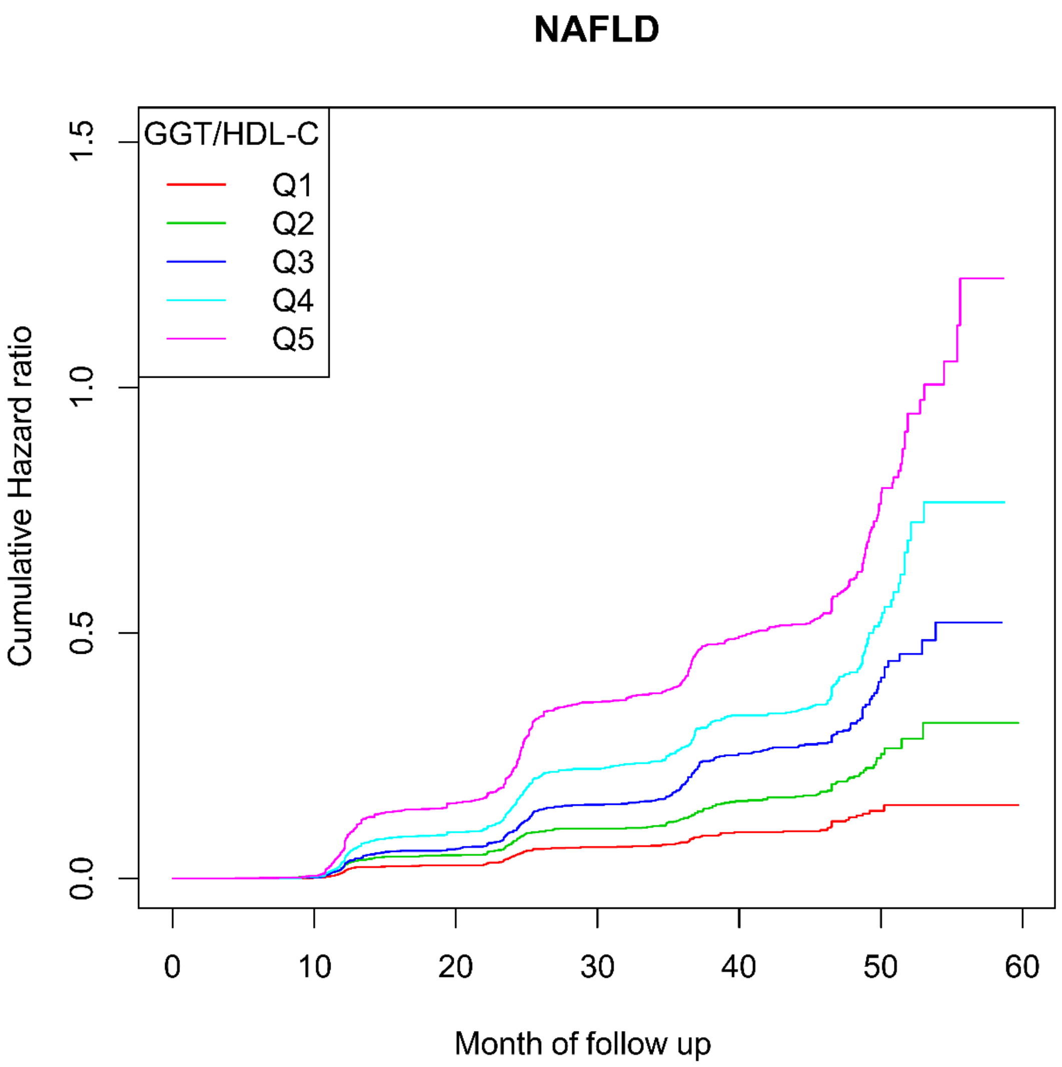

Supplementary Figure 1: Proportional hazards assumption checking. GGT: gamma-glutamyl transferase; HDL-C: high-density lipoprotein cholesterol.
