# Supplementary material for: Assessing the longitudinal association between the GGT/HDL-C ratio and NAFLD: a cohort study in a non-obese Chinese population
Source: BMC Gastroenterol. 2022 Dec 5;22:500. doi: 10.1186/s12876-022-02598-y (PMC9724423; doi:10.1186/s12876-022-02598-y)
Supplement: Supplementary file 4 — Additional file 4. Supplementary Table. [file 12876_2022_2598_MOESM4_ESM.docx]

Table S1: Collinearity diagnostics steps.

|  | VIF | | | | |
| --- | --- | --- | --- | --- | --- |
|  | Step 1 | Step 2 | Step 3 | Step 4 | Step 5 |
| GGT/HDL-C ratio | 11.4 | 11.4 | 11.4 | 1.4 | 1.4 |
| Sex | 1.1 | 1.1 | 1.1 | 1.1 | 1.1 |
| Age | 1.1 | 1.1 | 1.1 | 1.1 | 1.1 |
| ALP | 1.2 | 1.2 | 1.2 | 1.2 | 1.2 |
| GGT | 11.2 | 11.2 | 11.2 | NA | NA |
| ALT | 3.5 | 3.5 | 3.5 | 3.5 | 3.5 |
| AST | 3.6 | 3.6 | 3.6 | 3.6 | 3.6 |
| TP | 12006930 | NA | NA | NA | NA |
| ALB | 4625653.1 | 1.2 | 1.2 | 1.2 | 1.2 |
| GLB | 10588163.8 | 1.1 | 1.1 | 1.1 | 1.1 |
| TB | 2 | 2 | 2 | 2 | 2 |
| DBIL | 2.2 | 2.2 | 2.2 | 2.2 | 2.2 |
| BUN | 1.6 | 1.6 | 1.6 | 1.6 | 1.6 |
| Cr | 1.6 | 1.6 | 1.6 | 1.6 | 1.6 |
| UA | 1.7 | 1.7 | 1.7 | 1.7 | 1.7 |
| FPG | 1.2 | 1.2 | 1.2 | 1.2 | 1.2 |
| TC | 6.6 | 6.5 | 6.5 | 6.5 | NA |
| TG | 2.3 | 2.3 | 2.3 | 2.3 | 1.5 |
| HDL-C | 2.7 | 2.6 | 2.6 | 2.6 | 1.4 |
| LDL-C | 5.5 | 5.4 | 5.4 | 5 | 1.2 |
| Height | 105.2 | 105.2 | 1.4 | 1.4 | 1.4 |
| Weight | 244.1 | 244.1 | NA | NA | NA |
| BMI | 106.7 | 106.7 | 1.3 | 1.3 | 1.3 |
| SBP | 2.4 | 2.4 | 2.4 | 2.4 | 2.4 |
| DBP | 2 | 2 | 2 | 2 | 2 |

Abbreviations: VIF: Variance inflation factor; Other abbreviations as in Table 1.

Note: VIF = 1/(1-R^2^).

Table S2: Evaluation of GGT, HDL-C, GGT/HDL-C ratio and BMI for the diagnosis of NAFLD based on cross-sectional data.

|  | AUC | 95%CI low | 95%CI upp | Best threshold | Specificity | Sensitivity |
| --- | --- | --- | --- | --- | --- | --- |
| GGT | 0.7780 | 0.7750 | 0.7810 | 23.5000 | 0.6605 | 0.7750 |
| HDL-C | 0.6986 | 0.6952 | 0.7020 | 1.3350 | 0.6277 | 0.6749 |
| GGT/HDL-C ratio | 0.8050 | 0.8022 | 0.8078 | 18.7950 | 0.6993 | 0.7826 |
| BMI | 0.8164 | 0.8140 | 0.8188 | 22.1667 | 0.6624 | 0.8355 |

Abbreviations: AUC: area under the curve; other abbreviations as in Table ​1.
